# Urine-based PSMA detection for noninvasive prostate cancer diagnosis: recent advances, translational challenges, and future perspectives

**DOI:** 10.3389/fonc.2026.1833293

**Published:** 2026-04-29

**Authors:** Zunlong Jiao, Chenmei Deng, Jinying Hu, Sha Zhu

**Affiliations:** 1 Wuxi School of Medicine, Jiangnan University, Wuxi, China; 2 Department of Urology, Jiangnan University Medical Center/Wuxi No. 2 People’s Hospital, Wuxi, China

**Keywords:** CRISPR/Cas12a, nucleic acid aptamer, point-of-care testing, prostate cancer, PSMA, urine biomarker

## Abstract

Prostate cancer (PCa) is one of the most prevalent malignancies in men worldwide and continues to impose a substantial public health burden. Although prostate-specific antigen (PSA) testing remains the most widely used tool for PCa screening, its suboptimal specificity and moderate sensitivity, especially within the diagnostic gray zone of 4–10 ng/mL—frequently leads to unnecessary prostate biopsies, overdiagnosis, and overtreatment. Therefore, the development of sensitive, specific, and noninvasive diagnostic strategies has become a major priority in PCa management. Prostate-specific membrane antigen (PSMA), a type II transmembrane glycoprotein, is markedly overexpressed in PCa tissues and is closely associated with tumor aggressiveness, pathological progression, metastatic potential, and castration resistance. Owing to its strong disease association and established clinical relevance in molecular imaging and targeted therapy, PSMA has emerged as an attractive candidate for noninvasive diagnostic development. At the same time, urine has become an appealing liquid-biopsy substrate because it can be collected noninvasively, repeatedly, and with direct anatomical relevance to the prostate. Increasing evidence suggests that urinary PSMA-related analytes, including soluble PSMA, PSMA-positive extracellular vesicles (EVs), and associated transcripts, may provide a biological basis for noninvasive PCa detection. In this review, we summarize the biological rationale for PSMA as a urinary biomarker and critically examine recent advances in urinary PSMA detection technologies. Particular attention is given to antibody-based immunoassays and integrated biosensing systems that combine aptamer-mediated molecular recognition, CRISPR/Cas12a-assisted signal amplification, magnetic enrichment, and lateral flow assay (LFA) readout. We further discuss the major challenges hindering clinical implementation, including pre-analytical variability, urinary analyte heterogeneity, insufficient large-scale validation, and the limitations of single-biomarker strategies. Finally, future perspectives are outlined with emphasis on assay standardization, multimarker integration, and the development of clinically deployable point-of-care testing platforms. Collectively, urinary PSMA detection represents a promising but still emerging route toward more specific and noninvasive PCa diagnostics.

## 1 Introduction

PCa is among the most frequently diagnosed malignancies of the male genitourinary system and represents a growing global health burden. With population aging, increasing public health awareness, and broader implementation of screening programs, the incidence of PCa has continued to rise. Because disease stage at diagnosis is strongly associated with prognosis, early and accurate detection remains a critical determinant of clinical outcome. Localized PCa can often be effectively managed with curative treatment, whereas advanced or metastatic disease is associated with significantly higher morbidity and mortality. Therefore, improving early diagnostic strategies remains a major objective in PCa care (1, 2).

At present, serum PSA testing remains the cornerstone of PCa screening and early detection (3). However, PSA is organ-specific rather than tumor-specific, and elevated PSA levels may also occur in benign prostatic hyperplasia, prostatitis, urinary retention, or following urological procedures (4). This limitation is especially problematic in the diagnostic gray zone of 4–10 ng/mL, where PSA testing has suboptimal specificity and may lead to unnecessary biopsies, overdiagnosis, and overtreatment (5). Consequently, there is an urgent need to identify more specific biomarkers and to develop noninvasive assays with improved diagnostic accuracy.

Liquid biopsy has emerged as a promising strategy for noninvasive cancer detection because it allows repeated sampling and may dynamically reflect disease progression and tumor heterogeneity (6, 7). Among the available biological fluids, urine is particularly attractive for PCa detection because it can be obtained noninvasively, repeatedly, and at low cost, while also having direct anatomical relevance to the prostate. Proteins, exfoliated cells, EVs, cell-free nucleic acids, and other prostate-derived components can enter the urinary tract, making urine a valuable substrate for biomarker discovery and diagnostic assay development (8, 9). Urinary EVs and urine proteomes have been reported to reflect prostate-derived molecular information and show utility for diagnosis and risk stratification in PCa (8, 10).

PSMA is one of the most widely studied and clinically applied molecular targets in PCa. PSMA is expressed at low levels in normal prostate tissue but is markedly upregulated in PCa cells, particularly in high-grade, advanced, and castration-resistant disease (11, 12). Because of these properties, PSMA has been extensively exploited in molecular imaging and targeted therapy (13). More recently, growing attention has focused on its potential as a urinary biomarker, raising the possibility that PSMA-based detection may be translated into noninvasive diagnostic applications (14, 15). Urinary PSMA-positive EVs and urine-derived exosomal PSMA have both been reported as promising noninvasive biomarkers for PCa detection (14).

In this review, we aim to provide a comprehensive and critical overview of urinary PSMA detection for noninvasive PCa diagnosis. We first summarize the biological basis of PSMA as a urinary biomarker and its relevance in PCa. We then discuss recent advances in detection technologies, with particular emphasis on integrated biosensing strategies. Finally, we highlight key translational challenges and future perspectives, focusing on clinical applicability and standardization.

## 2 Biological basis of PSMA as a urinary biomarker

### 2.1 PSMA biology and its relevance to PCa

PSMA, also known as folate hydrolase 1 (FOLH1) or glutamate carboxypeptidase II, is a type II transmembrane glycoprotein with established overexpression in PCa (11, 12). Its expression is generally low in normal prostate tissue but becomes markedly elevated in malignant transformation and may further increase in aggressive, metastatic, and castration-resistant disease (12, 16).

Importantly, the same biological specificity that underlies PSMA-based theranostics also supports its exploration as a diagnostic biomarker (12, 13). Compared with many exploratory biomarkers that have limited clinical context, PSMA benefits from a strong translational foundation. Nevertheless, PSMA is not uniformly expressed in all PCas, and intertumoral as well as intratumoral heterogeneity may influence its diagnostic performance (12, 17). Therefore, while PSMA is highly promising, it should be viewed as a biologically enriched and clinically relevant marker rather than a universally sufficient one (17).

### 2.2 Why urine is a plausible matrix for PSMA-based testing

Urine is uniquely suited for PCa biomarker development because of its noninvasive accessibility and direct anatomical relevance to the genitourinary tract (6, 8). Prostate-derived material may enter urine through direct secretion, cellular shedding, or extracellular vesicle release (9). This makes urine a biologically meaningful matrix for detecting tumor-associated molecules.

The potential sources of urinary PSMA-related signals are likely heterogeneous (14, 18, 19). These may include soluble PSMA shed from tumor-associated membranes, PSMA-positive EVs, tumor-derived cellular debris, and PSMA-related transcripts such as FOLH1 mRNA (19, 20). This diversity strengthens the biological rationale for urinary detection but simultaneously complicates assay development, because different analytical platforms may target fundamentally different molecular forms under the same broad label of “urinary PSMA.”

Different detection strategies are inherently biased toward specific molecular forms of PSMA—soluble protein, EV-associated PSMA, or PSMA-related nucleic acids. For example, ELISA-based assays primarily target the soluble form, whereas immunomagnetic or aptamer-based enrichment approaches preferentially capture PSMA-positive EVs. CRISPR-based systems, on the other hand, typically rely on nucleic acid signal conversion strategies.

Each approach presents distinct advantages and limitations. Soluble PSMA detection is relatively straightforward but may suffer from low specificity, whereas EV-associated PSMA may better reflect tumor biology but requires more complex isolation procedures. Understanding these differences is critical for interpreting results and for selecting appropriate analytical platforms in future clinical applications.

### 2.3 Urinary PSMA in the context of other urinary biomarkers

Several urinary biomarkers have been investigated for the noninvasive detection of PCa, including PCA3, the TMPRSS2: ERG fusion transcript, exosomal RNAs, and various urinary miRNAs (21). These biomarkers have shown varying degrees of utility in early detection, risk stratification, and biopsy decision-making (8, 22). Compared with these markers, PSMA is particularly attractive because it is not only associated with molecular alterations in PCa but also strongly linked to disease aggressiveness and clinical progression (11–14, 18).

In this broader biomarker landscape, urinary PSMA offers several advantages. First, it is closely connected to PCa biology and tumor behavior. Second, it already has established clinical value in imaging and therapeutic targeting (12, 13). Third, it can be detected through a variety of assay formats, including protein-based, vesicle-based, and nucleic acid-assisted strategies (14, 18–20). However, urinary PSMA must still demonstrate robust analytical performance and clinically meaningful diagnostic value beyond currently available urine-based tests (8). Representative urinary biomarkers for PCa detection and their characteristics are summarized in **Table 1**. As shown, although several urinary biomarkers have demonstrated clinical utility, each has inherent limitations related to sensitivity, specificity, standardization, or workflow complexity. In this context, urinary PSMA represents a biologically relevant and technically versatile target, but its clinical value will ultimately depend on robust analytical validation and its ability to provide incremental diagnostic benefit beyond existing biomarkers.

**Table 1:** Representative urinary biomarkers for prostate cancer detection and their analytical characteristics.

Biomarker	Detection method	Clinical significance	Limitations	Representative references
PCA3	RT-PCR	Supports early detection and biopsy decision-making	Often requires prostate massage before urine collection	(21, 23)
TMPRSS2:ERG	qPCR	Improves diagnostic specificity, especially when combined with other markers	Limited sensitivity when used alone	(21, 23)
PSMA	ELISA/aptamer-based assay/integrated biosensor	Promising biomarker for noninvasive diagnosis and therapeutic stratification	Low abundance and incomplete standardization	(14, 18)
Exosomal RNA markers	RNA-based assays	May distinguish clinically significant disease from benign conditions	High cost and relatively complex workflow	(8, 22)
SelectMDx/multiplex gene panels	qPCR-based panels	Predicts the risk of clinically significant PCa	Interpretation may depend on algorithmic models	(21)
Urinary miRNAs	qPCR/sequencing	Potential biomarkers for diagnosis and prognosis	Poor inter-study standardization	(21)

## 3 Current landscape of urine-based PCa biomarkers

The field of urine-based PCa diagnostics has developed rapidly over the past decade (21). Commercial or late-stage tests based on transcripts, exosomes, and multiplex gene panels have demonstrated that urine can serve as a clinically practical source of tumor-associated information (24, 25). These tests are generally positioned as adjuncts to PSA and imaging, particularly in men with equivocal PSA levels or borderline clinical findings (24–26).

This context is important because urinary PSMA is not being developed in isolation (21). New PSMA-centered assays must ultimately demonstrate not only technical feasibility but also clinical value relative to established urinary biomarkers. In particular, they should ideally improve the detection of clinically significant PCa, reduce unnecessary biopsies, or enable more accessible point-of-care testing strategies. Thus, urinary PSMA should be viewed as part of a broader diagnostic ecosystem rather than as a standalone replacement for existing tests (24).

Importantly, the clinical utility of urinary PSMA detection should be evaluated in the context of established diagnostic tools. For example, PSA testing typically shows a sensitivity of approximately 70–80% but a specificity of only ~25–40% in the diagnostic gray zone (4–10 ng/mL), leading to substantial overdiagnosis (27). In contrast, urinary biomarkers such as PCA3 and SelectMDx have reported AUC values ranging from 0.68 to 0.85 in detecting clinically significant PCa (28).

Emerging studies on urinary PSMA detection, although still limited, have reported promising diagnostic performance. For instance, urinary exosomal PSMA assays have demonstrated AUC values approaching 0.80–0.90 in small cohorts, with improved specificity compared to PSA alone (18, 29). However, these findings remain preliminary and require validation in larger, well-characterized populations.

Therefore, the true translational value of urinary PSMA detection will depend on its ability to provide incremental diagnostic benefit beyond PSA and existing urinary biomarkers, particularly in clinically relevant populations such as patients within the PSA gray zone (30).

## 4 Recent advances in urinary PSMA detection technologies

### 4.1 Antibody-based immunoassays

Antibody-based immunoassays represent the most established and clinically accessible approach for protein detection and provide an important reference framework for evaluating emerging urinary PSMA detection technologies. These methods are widely used due to their standardized protocols, quantitative output, and compatibility with existing clinical laboratory infrastructure.

Representative studies have demonstrated that urinary PSMA can be detected using conventional immunoassay formats with moderate diagnostic performance. For example, Wang et al. reported that urinary exosomal PSMA achieved an AUC of approximately 0.82 for distinguishing PCa from benign conditions (18). Similarly, ELISA-based assays typically achieve limits of detection in the ng/mL range, although their performance in clinical settings remains constrained by matrix interference and low analyte abundance (31, 32).

In contrast, amplification-based biosensing platforms, particularly CRISPR/Cas12a-enabled systems, have demonstrated substantially improved analytical sensitivity under optimized experimental conditions. However, these systems remain largely at the proof-of-concept stage and lack sufficient clinical validation.

#### 4.1.1 Enzyme-linked immunosorbent assay

Enzyme-linked immunosorbent assay (ELISA) remains one of the most widely used methods for protein quantification. In the context of urinary PSMA, ELISA is attractive because it relies on well-established antibody–antigen interactions, provides quantitative output, and can be readily implemented in conventional laboratory settings. It also serves as a useful benchmark method for validating newer detection platforms (18, 20, 33, 34).

However, urinary PSMA analysis by ELISA faces several limitations. Urine is a complex biological matrix containing salts, metabolites, endogenous proteins, and other components that may interfere with antigen–antibody binding or reduce assay reproducibility. In addition, PSMA may be present at relatively low abundance in urine, necessitating sample concentration, purification, or enrichment steps. These additional procedures increase workflow complexity and may introduce variability (9, 35, 36). As a result, while ELISA remains valuable for analytical validation and exploratory studies, its direct suitability for rapid or decentralized clinical testing remains limited (34, 36).

#### 4.1.2 Chemiluminescence immunoassay and advanced affinity-based detection

Chemiluminescence immunoassay (CLIA) offers higher sensitivity, a broader dynamic range, and greater automation potential than conventional colorimetric ELISA. These features make CLIA theoretically attractive for detecting low-abundance urinary targets such as PSMA (37). In addition, advanced affinity-based systems incorporating nanoparticle amplification, fluorescence labels, or glycoform-specific recognition may further enhance analytical performance. A recent study describing an assay for aberrantly glycosylated urinary PSMA illustrates how analytically enriched PSMA subclasses may offer improved discrimination relative to total target measurement (20, 38).

Nevertheless, the utility of such methods in urinary PSMA detection remains limited by several factors, including instrumentation requirements, operational cost, and insufficient validation in real clinical samples (35, 38). Their translational value will therefore depend not only on increased sensitivity but also on whether they can detect clinically informative forms of urinary PSMA reproducibly and cost-effectively (38).

### 4.2 Integrated biosensing strategies for urinary PSMA detection

**Figure 1:**
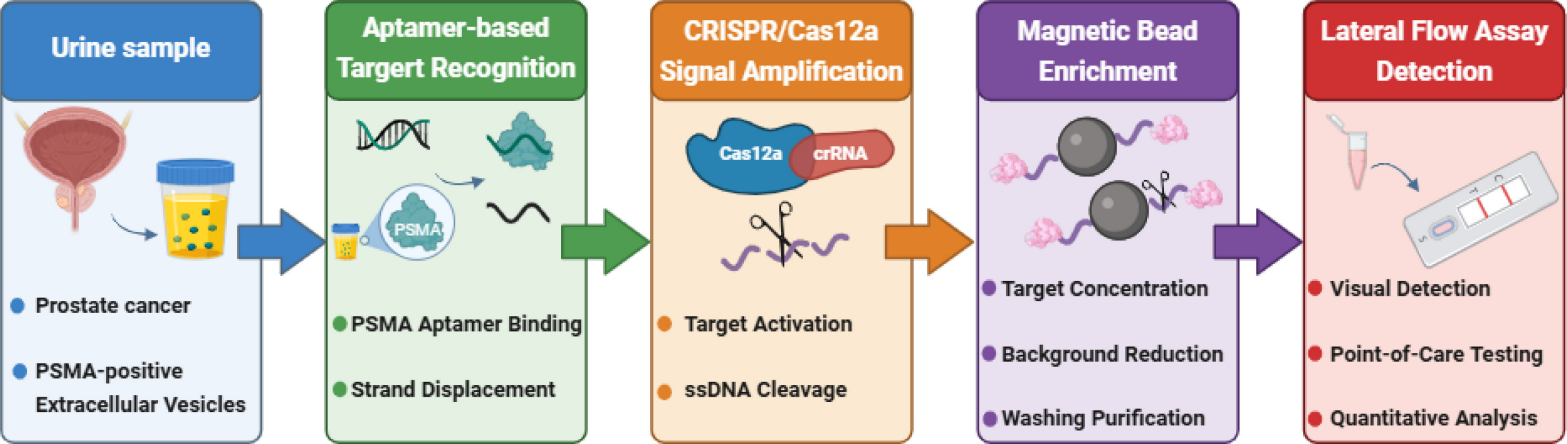
Integrated biosensing workflow for urine-based PSMA detection in noninvasive prostate cancer diagnosis. The platform combines aptamer-mediated molecular recognition, CRISPR/Cas12a-driven signal amplification, magnetic bead-based enrichment, and LFA readout, enabling sensitive, specific, and point-of-care-compatible detection of urinary PSMA.

Recent advances in urinary PSMA detection have increasingly moved beyond single-technology platforms toward integrated biosensing systems that combine molecular recognition, signal conversion, target enrichment, and portable readout within a unified analytical workflow (39). This trend reflects the practical reality that no individual technology is sufficient to simultaneously address all major analytical challenges associated with urinary PSMA detection, including low target abundance, matrix complexity, limited assay sensitivity, and the need for clinically deployable formats (18, 20, 35, 36, 39–41). As a result, hybrid platforms that incorporate aptamer-based recognition, CRISPR/Cas12a-mediated amplification, magnetic enrichment, and LFA readout have emerged as a particularly promising direction for next-generation noninvasive PCa diagnostics (40–43). As illustrated in **Figure 1**, these integrated platforms typically follow a modular workflow consisting of molecular recognition, signal amplification, target enrichment, and portable readout.

Within these integrated systems, aptamers often serve as the primary molecular recognition element (44, 45). Compared with antibodies, PSMA-targeting aptamers offer several advantages, including chemical synthesis, facile functional modification, batch-to-batch consistency, and direct compatibility with nucleic acid-responsive detection circuits (46). More importantly, aptamer–target binding can be rationally engineered to induce conformational switching or strand displacement, thereby converting protein recognition into a nucleic acid signal. This property makes aptamers especially suitable for incorporation into signal amplification platforms, where the binding event itself can act as the trigger for downstream molecular processing (46–48).

CRISPR/Cas12a represents a highly effective amplification engine for integrated biosensing platforms, primarily because target recognition activates its collateral ssDNA cleavage activity and enables strong signal multiplication through trans-cleavage of labeled reporters (49, 50). For protein analysis, this enzymatic property can be rationally coupled to aptamer-based molecular transduction, in which target binding induces strand release, strand displacement, or conformational exposure of a predesigned DNA activator that subsequently switches on Cas12a cleavage. This strategy is particularly well suited to urinary PSMA analysis, where low analyte abundance and matrix complexity often limit the performance of direct detection schemes. By introducing an additional programmable amplification layer downstream of target recognition, Cas12a-based designs can substantially improve assay sensitivity without sacrificing modularity or compatibility with diverse signal-output modalities (49–52). Several CRISPR/Cas12a-based biosensing platforms have achieved detection limits down to the pM–fM range under optimized experimental conditions (53, 54). However, these results are largely derived from proof-of-concept studies using spiked samples rather than real clinical cohorts.

Magnetic enrichment provides a complementary upstream module that improves the analytical performance of urine-based assays by increasing target recovery, reducing nonspecific background, and simplifying post-capture washing and purification steps (14, 32). In practice, magnetic particles can be functionalized with antibodies, aptamers, or nucleic-acid probes to selectively isolate soluble biomarkers, EVs, or reporter-bearing complexes from urine (8, 14, 32). This is especially valuable in urinary diagnostics, where biomarker concentrations are low and pre-analytical variability is high. Notably, PSMA-positive EVs have already been isolated from urine by immunomagnetic approaches, supporting the feasibility of PSMA-directed vesicle enrichment as a PCa liquid-biopsy strategy (14). In parallel, recent urinary proteomic evidence indicates that extracellular-vesicle fractions can preserve prostate-derived molecular signatures and may provide biologically more informative material than unfractionated urine for downstream tumor-associated analysis (8, 9).

For practical deployment, LFA-based and other portable readout formats are particularly valuable because they enable rapid, low-cost, and user-friendly detection (55, 56). Traditional LFAs, however, often lack the sensitivity required for low-abundance tumor biomarkers, and this limitation can be partially overcome in integrated systems by upstream amplification and enrichment modules (56, 57). For example, aptamer recognition may first generate a nucleic acid trigger, CRISPR/Cas12a may then amplify the signal through collateral reporter cleavage, magnetic particles may concentrate the relevant analytes or signal complexes, and the final output may be visualized on an LFA strip (57–59). Such multistage designs preserve the operational simplicity of strip-based detection while markedly improving analytical performance (57–59). Similar strategies may also be adapted to fluorescence, electrochemical, or microfluidic readout formats, depending on the intended clinical setting (40, 60, 61).

Taken together, these integrated biosensing strategies represent a major evolution in urinary PSMA assay design. Rather than relying on a single recognition or readout mechanism, they exploit the complementary strengths of different technologies: aptamers provide selective and programmable recognition, CRISPR/Cas12a enables ultrasensitive signal amplification, magnetic particles improve analyte enrichment and matrix cleanup, and LFA supports portable point-of-care readout (32, 40, 56, 62). This modular architecture is highly attractive for translational development because each component can be independently optimized while remaining compatible with the overall workflow (40, 56, 62).

Nevertheless, several important challenges must be addressed before such integrated systems can be translated into routine clinical practice (63, 64). The combination of multiple functional modules may increase assay complexity, reagent instability, background leakage, and manufacturing variability (63, 65). Moreover, strong analytical performance in proof-of-concept studies does not necessarily guarantee reproducibility in real clinical urine samples. Therefore, future development should focus not only on maximizing sensitivity, but also on simplifying workflow design, improving matrix tolerance, enhancing batch consistency, and validating performance in large prospective patient cohorts (63–66).

Despite their conceptual advantages, integrated biosensing platforms remain at an early developmental stage and face several nontrivial challenges. First, the integration of multiple functional modules increases system complexity, which may compromise robustness and reproducibility (67).Second, batch-to-batch variability in reagents such as aptamers, enzymes, and nanoparticles can introduce significant analytical variability (68). Third, many reported studies rely on spiked samples or small clinical cohorts, and their performance in heterogeneous real-world urine samples remains insufficiently validated (69).

Importantly, there is a tendency in the literature to overstate analytical performance without adequately addressing practical constraints such as workflow complexity, assay time, and operator dependency (70). Therefore, distinguishing clearly between proof-of-concept demonstrations and clinically validated technologies is essential for accurately assessing the translational potential of these platforms (69).

Taken together, integrated biosensing strategies represent a significant advancement in urinary PSMA assay design by combining complementary technologies to overcome individual limitations. However, their translational potential should be interpreted with caution (71) Despite their conceptual advantages, these systems remain at an early developmental stage. The integration of multiple functional modules increases assay complexity and may compromise reproducibility (72). In addition, batch-to-batch variability in key components such as aptamers, enzymes, and nanomaterials introduces further uncertainty (72). Importantly, many reported studies emphasize analytical sensitivity without adequately addressing practical constraints, including workflow complexity, assay time, and operator dependence (73). Therefore, it is essential to distinguish clearly between proof-of-concept demonstrations and clinically validated technologies (71). Future studies should prioritize standardized evaluation, simplified workflows, and large-scale clinical validation to define the real-world applicability of these platforms.

## 5 Comparative advantages and limitations of current approaches

A comparison of the major technology classes for urinary PSMA detection is provided in **Table 2**. Importantly, these platforms differ not only in their underlying analytical principles but also in quantitative performance, clinical applicability, operational complexity, and translational readiness. As summarized, current analytical approaches can be broadly categorized into conventional immunoassays, advanced affinity-based detection systems, and integrated biosensing strategies (76). To further delineate these differences, **Table 3** provides a quantitative and clinically oriented comparison, including key parameters such as limit of detection, dynamic range, time to result, and clinical role, thereby enabling a more comprehensive evaluation of their translational potential.

**Table 2:** Comparison of major technology classes for urinary PSMA detection.

Platform	Core principle	Major strengths	Limitations	Clinical context	Translational potential	Representative references
ELISA/conventional immunoassay	Antibody–antigen binding	Familiar, quantitative, relatively standardized	Matrix interference, low analyte abundance, laboratory dependence	Validation/research	Useful for validation, limited for POCT	(18, 32)
CLIA/advanced affinity assay	Enhanced immunodetection with luminescent or nanoparticle-assisted readout	High sensitivity, broader dynamic range	Cost, instrumentation, limited urinary validation	Hospital lab	Promising in centralized settings	(8, 32, 74)
Integrated biosensing platform	Aptamer recognition + CRISPR amplification + magnetic enrichment + LFA/portable readout	High sensitivity, modularity, point-of-care potential	Multicomponent complexity, reproducibility challenges	POCT/screening	Highly promising but still early-stage	(8, 9, 32, 39, 75)

**Table 3:** Comparative overview of urinary PSMA detection platforms.

Platform	Target analyte	Limit of detection	Dynamic range	Time to result	Major strengths	Key limitations	Clinical role	Translational stage
ELISA	Soluble PSMA protein	1–10pM	10pM–100nM	2–4h	Quantitative, standardized	Matrix interference, low sensitivity	Reference method	Clinically established
CLIA	Soluble PSMA/glycoforms	10–100fM	100fM–10nM	1–2h	High sensitivity, automated	Cost, instrumentation	Central lab diagnostics	Early clinical adoption
EV-based assays	PSMA+ EVs (protein/RNA)	100fM–10pM	pM–nM	4–24h	High biological relevance	Isolation complexity	Biomarker discovery	Emerging
Aptamer–CRISPR biosensor	Nucleic acid-converted signal	1–100fM	fM–nM	30–90min	Ultra-sensitive, modular	Limited clinical validation	POCT potential	Proof-of-concept
Integrated biosensing platforms	Multi-module hybrid signal	1–100fM	fM–nM	30–120min	Amplification + enrichment	Reproducibility issues	Next-gen POCT	Early translational

Data summarized from representative studies on urinary PSMA detection and related biosensing platforms (19, 21, 30–33, 37, 49, 54–58).

Conventional immunoassays, such as ELISA, are widely used due to their established protocols, quantitative output, and relatively high reproducibility. These methods are particularly valuable as reference techniques for analytical validation. However, their performance in urinary PSMA detection is often limited by matrix interference, low analyte abundance, and the need for laboratory-based instrumentation, which restricts their applicability in decentralized or point-of-care settings (32–34). As summarized in **Table 3**, ELISA typically exhibits detection limits in the ng/mL range and relatively long assay times, which further constrain its utility in rapid clinical decision-making. Therefore, ELISA is best positioned as a reference and benchmarking tool rather than a frontline diagnostic modality, particularly in the context of decentralized testing.

Advanced affinity-based assays, including chemiluminescence immunoassays and nanoparticle-enhanced detection systems, offer improved sensitivity and broader dynamic range. These approaches may enable the detection of low-abundance PSMA species or specific molecular subtypes, such as aberrantly glycosylated PSMA. Nevertheless, their clinical translation is constrained by high cost, complex instrumentation, and limited validation in large-scale urinary studies (33, 36). Compared with ELISA, these methods demonstrate improved analytical sensitivity (typically in the pg/mL range, as shown in **Table 3**), but their dependence on centralized laboratory infrastructure limits their applicability in decentralized clinical settings. As indicated in **Table 3**, although sensitivity is enhanced, operational complexity and cost remain significant barriers to widespread clinical adoption. Accordingly, these approaches are more suitable for high-throughput hospital-based diagnostics rather than point-of-care applications.

In contrast, integrated biosensing platforms that combine aptamer-mediated recognition, CRISPR/Cas12a-based signal amplification, magnetic enrichment, and portable readout formats (e.g., lateral flow assays) have emerged as a promising next-generation strategy. These systems are designed to address multiple analytical challenges simultaneously, including low target abundance, complex sample matrices, and the need for rapid and user-friendly detection. By leveraging the complementary strengths of each module, integrated platforms can achieve enhanced sensitivity, modularity, and point-of-care applicability (37, 49, 58). Importantly, these systems are uniquely positioned for future POCT deployment, provided that their robustness and reproducibility can be adequately addressed. Notably, as summarized in **Table 3**, these platforms can achieve detection limits down to the pM–fM range with significantly reduced assay time (typically within 30–120 min), highlighting their potential for rapid and highly sensitive detection. This performance advantage is clearly illustrated in **Table 3**, where integrated platforms outperform conventional immunoassays in analytical sensitivity and turnaround time. Importantly, these features make integrated biosensing systems particularly attractive for future point-of-care testing applications and decentralized diagnostic scenarios. However, it remains unclear whether such improvements in analytical sensitivity translate into meaningful clinical benefits, such as improved detection of clinically significant prostate cancer or reduction of unnecessary biopsies.

However, despite their conceptual advantages, integrated systems remain in an early stage of development. Their multicomponent nature may introduce challenges related to assay complexity, reagent stability, background signal leakage, and batch-to-batch variability. In addition, robust clinical validation in large and well-defined patient cohorts is still lacking. Therefore, while integrated biosensing strategies hold considerable promise, further optimization and standardization are required before they can be translated into routine clinical practice. Furthermore, the discrepancy between analytical performance under controlled experimental conditions and real-world clinical performance remains a critical unresolved issue. These limitations collectively represent major barriers to clinical translation and large-scale implementation.

Overall, as highlighted in **Table 2**, no single analytical platform currently satisfies all clinical and operational requirements. Conventional immunoassays provide robustness and standardization but lack sensitivity and portability; advanced affinity-based assays improve analytical performance but remain infrastructure-dependent; whereas integrated biosensing platforms offer superior sensitivity and point-of-care testing potential but require further validation and simplification. Future development should therefore focus not only on improving analytical sensitivity but also on enhancing robustness, reproducibility, workflow simplicity, and clinical relevance. Importantly, standardized head-to-head comparisons across different detection platforms under harmonized experimental conditions will be essential to define the clinical positioning of urinary PSMA assays and to determine whether they provide meaningful incremental value over existing diagnostic strategies. In particular, future studies should evaluate these platforms in clinically relevant populations, such as patients within the PSA gray zone, where improved specificity is most urgently needed.

## 6 Major challenges in urinary PSMA detection

### 6.1 Pre-analytical variability and lack of standardization

One of the major barriers to urinary PSMA assay development is the absence of standardized pre-analytical procedures. Variations in urine collection timing, first-void versus random sampling, prostate massage or digital rectal examination prior to collection, centrifugation protocols, storage conditions, and freeze–thaw cycles may all influence analyte recovery and assay performance (77–82). Without harmonized procedures, inter-study comparison remains difficult and clinical reproducibility is compromised (77–81).

To address these challenges, several standardization strategies should be considered (77). First, urine collection protocols should be harmonized, including recommendations on first-void versus random sampling, timing relative to digital rectal examination, and patient preparation (83). Second, sample processing procedures such as centrifugation speed, filtration, and storage conditions should be standardized to minimize variability in analyte recovery (84). Third, normalization strategies—such as creatinine normalization, total protein normalization, or extracellular vesicle quantification—should be incorporated to account for inter-sample variability (77).

In addition, the adoption of reporting guidelines, similar to MISEV standards for EVs, may help improve reproducibility and comparability across studies (69). Establishing such frameworks will be critical for advancing urinary PSMA detection toward clinical implementation (77).

### 6.2 Analyte heterogeneity

Urinary PSMA is not a single molecular entity. Different platforms may detect soluble PSMA protein, PSMA-associated EVs, or PSMA-related transcripts. These analytes differ in biological origin, abundance, stability, and potential clinical significance. This lack of analytical uniformity complicates cross-study comparison and hinders the establishment of clinically meaningful thresholds (80, 81).

### 6.3 Insufficient clinical validation

Most current studies on urinary PSMA detection remain exploratory or proof-of-concept in nature (18, 81, 85). Sample sizes are often limited, patient cohorts are heterogeneous, and external validation is rare. In particular, there is insufficient evidence regarding the performance of urinary PSMA assays in clinically important populations such as men with PSA values in the gray zone, patients with clinically significant disease, or those undergoing longitudinal monitoring. Large prospective multicenter studies are therefore needed (86, 87).

### 6.4 Limitations of single-biomarker strategies

PCa is a biologically heterogeneous disease, and no single biomarker is likely to capture its full complexity. Although PSMA is biologically compelling, its standalone diagnostic performance may be insufficient in certain settings. Multimarker approaches that combine PSMA with PCA3, TMPRSS2:ERG, urinary miRNAs, extracellular vesicle signatures, or imaging findings may improve diagnostic accuracy and clinical relevance (9, 86–90).

### 6.5 Clinical translation considerations

Beyond analytical performance, successful clinical translation of urinary PSMA assays requires careful consideration of regulatory approval, cost-effectiveness, scalability, and integration into existing clinical workflows.

From a regulatory perspective, diagnostic assays must demonstrate analytical validity, clinical validity, and clinical utility in accordance with guidelines from agencies such as the FDA or EMA. Currently, most urinary PSMA detection platforms remain at the proof-of-concept stage and have not yet undergone rigorous regulatory evaluation (71).

Cost and scalability are also critical factors (70). While integrated biosensing platforms offer high sensitivity, their multicomponent design may increase manufacturing complexity and cost, potentially limiting widespread adoption (67). Simplified assay formats with minimal instrumentation requirements will be more compatible with point-of-care deployment (73).

In terms of clinical workflow integration, urinary PSMA assays are most likely to function as adjunctive tools rather than standalone diagnostics. For example, they may be incorporated into decision-making pathways alongside PSA testing, MRI findings, and other urinary biomarkers to improve biopsy selection and risk stratification (91, 92).

Therefore, future development should prioritize not only analytical innovation but also real-world feasibility, regulatory readiness, and clinical usability.

## 7 Future perspectives

The future development of urinary PSMA detection should focus on both analytical optimization and clinical translation. First, standardized protocols for urine collection, pretreatment, storage, and normalization are urgently needed to improve reproducibility and comparability across studies (90). Second, assay development should emphasize not only analytical sensitivity but also matrix tolerance, ease of operation, batch consistency, and cost-effectiveness (93).Third, future studies should be designed around clinically relevant questions, especially whether urinary PSMA can help distinguish clinically significant PCa from indolent disease and reduce unnecessary biopsies in patients with equivocal PSA results. In this regard, multimarker integration may represent one of the most promising directions. PSMA-based detection could be combined with transcriptomic markers, exosomal biomarkers, urinary proteomics, or clinical risk models to generate more robust diagnostic frameworks (23, 94).

From a technological standpoint, hybrid systems that integrate aptamer recognition, CRISPR amplification, magnetic enrichment, and LFA or microfluidic readout may eventually yield clinically deployable point-of-care devices. However, for such systems to become truly viable, they must demonstrate not only excellent analytical performance under controlled conditions but also reproducibility, scalability, operational simplicity, and diagnostic value in real-world patient cohorts.

Ultimately, the field must move beyond elegant proof-of-concept biosensors toward rigorously validated assays aligned with genuine clinical decision-making (81, 87). This transition will require close collaboration among molecular biologists, analytical chemists, biomedical engineers, and clinicians.

## 8 Conclusion

Urinary PSMA detection represents a scientifically compelling and clinically relevant strategy for the noninvasive diagnosis of PCa. Its appeal arises from the convergence of three major factors: the strong biological relevance of PSMA in PCa, the practical advantages of urine as a liquid-biopsy substrate, and rapid advances in biosensing technologies capable of detecting low-abundance analytes with increasing sensitivity.

Among currently developing approaches, integrated biosensing systems that combine aptamer-mediated recognition, CRISPR/Cas12a-assisted amplification, magnetic enrichment, and portable readout have emerged as particularly promising. These hybrid strategies offer a pathway toward more sensitive, specific, and clinically deployable urinary PSMA assays.

Nevertheless, significant obstacles remain before urinary PSMA testing can be translated into routine clinical practice. These include pre-analytical variability, analyte heterogeneity, limited large-scale validation, and the need to demonstrate clear incremental value over existing biomarkers and diagnostic workflows. With continued technological refinement, rigorous clinical validation, and deeper integration into multimodal diagnostic frameworks, urinary PSMA-based assays may ultimately become valuable tools for PCa screening, biopsy triage, risk stratification, and disease monitoring.

## References

[1] Sung H Ferlay J Siegel RL Laversanne M Soerjomataram I Jemal A . Global cancer statistics 2020: GLOBOCAN estimates of incidence and mortality worldwide for 36 cancers in 185 countries. CA Cancer J Clin. (2021) 71:209–49. doi: 10.3322/caac.21660. PMID: 33538338

[2] Carlsson SV Vickers AJ . Screening for prostate cancer. Med Clin North Am. (2020) 104:1051–62. doi: 10.1016/j.mcna.2020.08.007. PMID: 33099450 PMC8287565

[3] Ilic D Djulbegovic M Jung JH Hwang EC Zhou Q Cleves A . Prostate cancer screening with prostate-specific antigen (PSA) test: a systematic review and meta-analysis. BMJ. (2018) 362:k3519. doi: 10.1136/bmj.k3519. PMID: 30185521 PMC6283370

[4] Saini S . PSA and beyond: alternative prostate cancer biomarkers. Cell Oncol (Dordr). (2016) 39:97–106. doi: 10.1007/s13402-016-0268-6. PMID: 26790878 PMC4821699

[5] Guo E Xu L Zhang D Zhang J Zhang X Bai X . Diagnostic performance of MRI in detecting prostate cancer in patients with prostate-specific antigen levels of 4-10 ng/mL: a systematic review and meta-analysis. Insights Imaging. (2024) 15:147. doi: 10.1186/s13244-024-01699-4. PMID: 38886256 PMC11183000

[6] Crocetto F Musone M Chianese S Conforti P Digitale Selvaggio G Caputo VF . Blood and urine-based biomarkers in prostate cancer: Current advances, clinical applications, and future directions. J Liq Biopsy. (2025) 9:100305. doi: 10.1016/j.jlb.2025.100305. PMID: 40606769 PMC12221370

[7] Robaczyńska J Maj M Kiljańczyk A Pastuszek B Reducha E Nurkiewicz A . Epigenetic and liquid biopsy biomarkers in prostate cancer: Bridging tumor heterogeneity and clinical implementation. Cancers (Basel). (2026) 18. doi: 10.3390/cancers18030389, PMID: 41681860 PMC12896875

[8] Smith SF Brewer DS Hurst R Cooper CS . Applications of urinary extracellular vesicles in the diagnosis and active surveillance of prostate cancer. Cancers (Basel). (2024) 16. doi: 10.3390/cancers16091717. PMID: 38730670 PMC11083542

[9] Khoo A Govindarajan M Qiu Z Liu LY Ignatchenko V Waas M . Prostate cancer reshapes the secreted and extracellular vesicle urinary proteomes. Nat Commun. (2024) 15:5069. doi: 10.1038/s41467-024-49424-5. PMID: 38871730 PMC11176296

[10] Frantzi M Morillo AC Lendinez G Blanca A Lopez Ruiz D Parada J . Validation of a urine-based proteomics test to predict clinically significant prostate cancer: Complementing mpMRI pathway. Pathobiology. (2025) 92:99–108. doi: 10.1159/000542465. PMID: 39527943

[11] Bakht MK Beltran H . Biological determinants of PSMA expression, regulation and heterogeneity in prostate cancer. Nat Rev Urol. (2025) 22:26–45. doi: 10.1038/s41585-024-00900-z. PMID: 38977769 PMC11841200

[12] Corpetti M Müller C Beltran H de Bono J Theurillat J-P . Prostate-specific membrane antigen–targeted therapies for prostate cancer: Towards improving therapeutic outcomes. Eur Urol. (2024) 85:193–204. doi: 10.1016/j.eururo.2023.11.018. PMID: 38104015

[13] Sartor O de Bono J Chi KN Fizazi K Herrmann K Rahbar K . Lutetium-177-PSMA-617 for metastatic castration-resistant prostate cancer. N Engl J Med. (2021) 385:1091–103. doi: 10.1056/nejmoa2107322. PMID: 34161051 PMC8446332

[14] Allelein S Aerchlimann K Rösch G Khajehamiri R Kölsch A Freese C . Prostate-specific membrane antigen (PSMA)-positive extracellular vesicles in urine-a potential liquid biopsy strategy for prostate cancer diagnosis? Cancers (Basel). (2022) 14. doi: 10.3390/cancers14122987. PMID: 35740652 PMC9221222

[15] Permpoka K Kaewarsa P Paekoh W Opanuraks J Laiwattanapaisal W Estrela P . A capacitance biosensor for prostate cancer detection via normalised urinary extracellular vesicles. Biosens Bioelectron. (2025) 288:117791. doi: 10.2139/ssrn.5238262 40684732

[16] Wang H Li G Zhao J Eiber M Tian R . Current status of PSMA-targeted imaging and therapy. Front Oncol. (2023) 13:1230251. doi: 10.3389/fonc.2023.1230251. PMID: 38264741 PMC10803481

[17] Mulati Y Shen Q Tian Y Chen Y Yao K Yu W . Characterizing PSMA heterogeneity in prostate cancer and identifying clinically actionable tumor associated antigens in PSMA negative cases. Sci Rep. (2025) 15:23902. doi: 10.1038/s41598-025-06393-z. PMID: 40615466 PMC12227638

[18] Wang CB Chen SH Zhao L Jin X Chen X Ji J . Urine-derived exosomal PSMA is a promising diagnostic biomarker for the detection of prostate cancer on initial biopsy. Clin Transl Oncol. (2023) 25:758–67. doi: 10.1007/s12094-022-02983-9. PMID: 36266386

[19] Žvirblė M Vaicekauskaitė I Survila ŽChecktae Bosas P Dobrovolskienė N Mlynska A . Liquid-based diagnostic panels for prostate cancer: The synergistic role of soluble PD-L1, PD-1, and mRNA biomarkers. Int J Mol Sci. (2025) 26. doi: 10.3390/ijms26020704, PMID: 39859417 PMC11765789

[20] Khan M Islam MK Taimen P Boström PJ Lamminmäki U Leivo J . Aberrantly glycosylated PSMA in urine as a potential marker for prostate cancer. Clin Chim Acta. (2026) 582:120790. doi: 10.1016/j.cca.2025.120790. PMID: 41429247

[21] Liu Y Hatano K Nonomura N . Liquid biomarkers in prostate cancer diagnosis: Current status and emerging prospects. World J Mens Health. (2025) 43:8–27. doi: 10.5534/wjmh.230386. PMID: 38772530 PMC11704174

[22] Yu J Yu C Jiang K Yang G Yang S Tan S . Unveiling potential: urinary exosomal mRNAs as non-invasive biomarkers for early prostate cancer diagnosis. BMC Urol. (2024) 24:163. doi: 10.1186/s12894-024-01540-6. PMID: 39090720 PMC11292860

[23] Warli SM Warli MH Prapiska FF . PCA3 and TMPRSS2: ERG urine level as diagnostic biomarker of prostate cancer. Res Rep Urol. (2023) 15:149–55. doi: 10.2147/rru.s401131. PMID: 37181497 PMC10167967

[24] Tosoian JJ Zhang Y Xiao L Xie C Samora NL Niknafs YS . Development and validation of an 18-gene urine test for high-grade prostate cancer. JAMA Oncol. (2024) 10:726–36. doi: 10.1001/jamaoncol.2024.0455. PMID: 38635241 PMC11190811

[25] Margolis E Brown G Partin A Carter B McKiernan J Tutrone R . Predicting high-grade prostate cancer at initial biopsy: clinical performance of the ExoDx (EPI) Prostate Intelliscore test in three independent prospective studies. Prostate Cancer Prostatic Dis. (2022) 25:296–301. doi: 10.1038/s41391-021-00456-8. PMID: 34593984 PMC9184274

[26] Robinson HS Lee SS Barocas DA Tosoian JJ . Evaluation of blood and urine based biomarkers for detection of clinically-significant prostate cancer. Prostate Cancer Prostatic Dis. (2025) 28:45–55. doi: 10.1038/s41391-024-00840-0. PMID: 38858447 PMC12143304

[27] Li G Tholance Y Mallouk N Waeckel L Flandrin P Bali B . Quantification of urinary exosomal prostate-specific antigen for the diagnosis of prostate cancer using clinical laboratory-based techniques: Protocol for a case-control study. JMIR Res Protoc. (2024) 13:e63551. doi: 10.2196/63551. PMID: 39024018 PMC11425016

[28] Gil-Sousa D Oliveira-Reis D Tavares C Teves F Osório L Gomes M . Prostate biopsy evolution and the need for repeat biopsy - The role of image and new prostate cancer biomarkers. Arch Esp Urol. (2019) 72:677–89. 31475679

[29] Gan J Zeng X Wang X Wu Y Lei P Wang Z . Effective diagnosis of prostate cancer based on mRNAs from urinary exosomes. Front Med. (2022) 9:736110. doi: 10.3389/fmed.2022.736110. PMID: 35402423 PMC8983915

[30] Soeterik TFW Wu X Van den Bergh RCN Kesch C Zattoni F Falagario U . Personalised prostate cancer diagnosis: Evaluating biomarker-based approaches to reduce unnecessary magnetic resonance imaging and biopsy procedures. Eur Urol Open Sci. (2025) 75:106–19. doi: 10.1016/j.euros.2025.03.006. PMID: 40291786 PMC12032181

[31] Kawakami K Fujita Y Kato T Horie K Koie T Umezawa K . Diagnostic potential of serum extracellular vesicles expressing prostate-specific membrane antigen in urologic Malignancies. Sci Rep. (2021) 11:15000. doi: 10.1038/s41598-021-94603-9. PMID: 34294841 PMC8298409

[32] Wang N Yuan S Fang C Hu X Zhang Y-S Zhang L-L . Nanomaterials-based urinary extracellular vesicles isolation and detection for non-invasive auxiliary diagnosis of prostate cancer. Front Med. (2022) 8:800889. doi: 10.3389/fmed.2021.800889. PMID: 35096890 PMC8795515

[33] Wu Y Wang Y Huang Z Liu Q . Recent advances in analysis technology for detection of prostate cancer biomarkers. Microchem J. (2023) 190:108740. doi: 10.1016/j.microc.2023.108740. PMID: 38826717

[34] Huang Y Mao J Li X . Emerging biomarkers in prostate cancer diagnosis and treatment: Insights into genetic, RNA and metabolic markers (review). Int J Oncol. (2026) 68. doi: 10.3892/ijo.2025.5828. PMID: 41347816 PMC12716904

[35] Jordaens S Zwaenepoel K Tjalma W Deben C Beyers K Vankerckhoven V . Urine biomarkers in cancer detection: A systematic review of preanalytical parameters and applied methods. Int J Cancer. (2023) 152:2186–205. doi: 10.1002/ijc.34434. PMID: 36647333

[36] Safari F Kehelpannala C Safarchi A Batarseh AM Vafaee F . Biomarker reproducibility challenge: A review of non-nucleotide biomarker discovery protocols from body fluids in breast cancer diagnosis. Cancers (Basel). (2023) 15. doi: 10.3390/cancers15102780. PMID: 37345117 PMC10216598

[37] Zhao L Sun L Chu X . Chemiluminescence immunoassay. TrAC Trends Anal Chem. (2009) 28:404–15. doi: 10.1016/j.trac.2008.12.006. PMID: 38826717

[38] Subramani IG Ayub RM Gopinath SCB Perumal V Fathil MFM Md Arshad MK . Lectin bioreceptor approach in capacitive biosensor for prostate-specific membrane antigen detection in diagnosing prostate cancer. J Taiwan Inst Chem Eng. (2021) 120:9–16. doi: 10.1016/j.jtice.2021.03.004. PMID: 38826717

[39] Amin-Sadrabadi E Omidfar K . Non-invasive urine-based detection of PSMA-positive exosomes using a dual-aptamer electrochemical aptasensor for prostate cancer diagnosis. Mikrochim Acta. (2026) 193:77. doi: 10.1007/s00604-025-07761-2. PMID: 41505006

[40] Du K Zeng Q Jiang M Hu Z Zhou M Xia K . CRISPR/Cas12a-based biosensing: Advances in mechanisms and applications for nucleic acid detection. Biosensors [Internet]. (2025) 15:360. doi: 10.3390/bios15060360. PMID: 40558442 PMC12190445

[41] Guo Y Ren H Wang H Duan X Qi S Yang X . Aptamer-CRISPR/Cas12a-based lateral flow technique for visualized rapid detection of endogenous damage factor Neu5Gc in red meat. Foods. (2025) 14. doi: 10.3390/foods14162879. PMID: 40870790 PMC12385705

[42] Cheng HP Yang TH Wang JC Chuang HS . Recent trends and innovations in bead-based biosensors for cancer detection. Sensors (Basel). (2024) 24. doi: 10.3390/s24092904. PMID: 38733011 PMC11086254

[43] Liu G . Advancing CRISPR/Cas biosensing with integrated devices. ACS Sens. (2025) 10:575–6. doi: 10.1021/acssensors.5c00330. PMID: 40017406

[44] Campos-Fernández E Oliveira Alqualo N Moura Garcia LC Coutinho Horácio Alves C Ferreira Arantes Vieira TD Caixeta Moreira D . The use of aptamers in prostate cancer: A systematic review of theranostic applications. Clin Biochem. (2021) 93:9–25. doi: 10.1016/j.clinbiochem.2021.03.014. PMID: 33794195

[45] Cruz-Hernández CD Rodríguez-Martínez G Cortés-Ramírez SA Morales-Pacheco M Cruz-Burgos M Losada-García A . Aptamers as theragnostic tools in prostate cancer. Biomolecules. (2022) 12. doi: 10.3390/biom12081056. PMID: 36008950 PMC9406110

[46] Mahboobi M Najafi A Kooshki H Kheirandish M Soofian SE Sedighian H . Novel approaches for detection and targeted therapy of prostate cancer using antibodies, aptamers, and nanobodies. Mater Adv. (2025) 6:8816–38. doi: 10.1039/d5ma00933b

[47] Lee H Xie T Kang B Yu X Schaffter SW Schulman R . Plug-and-play protein biosensors using aptamer-regulated *in vitro* transcription. Nat Commun. (2024) 15:7973. doi: 10.1038/s41467-024-51907-4. PMID: 39266511 PMC11393120

[48] Lu B Lin S Lang Z Yin Q Cao H . Aptamer probe-assisted strand displacement-CRISPR/Cas12a biosensor for ultrasensitive detection of AFB1. J Agric Food Chem. (2025) 73:20500–7. doi: 10.1021/acs.jafc.5c05775. PMID: 40743400

[49] Chen JS Ma E Harrington LB Da Costa M Tian X Palefsky JM . CRISPR-Cas12a target binding unleashes indiscriminate single-stranded DNase activity. Science. (2018) 360:436–9. doi: 10.1126/science.aar6245. PMID: 29449511 PMC6628903

[50] Su W Li J Ji C Chen C Wang Y Dai H . CRISPR/Cas systems for the detection of nucleic acid and non-nucleic acid targets. Nano Res. (2023) 16(7):9940–53. doi: 10.1007/s12274-023-5567-4. PMID: 37359078 PMC10026200

[51] Yang R Zhao L Wang X Kong W Luan Y . Recent progress in aptamer and CRISPR-Cas12a based systems for non-nucleic target detection. Crit Rev Anal Chem. (2024) 54:2670–87. doi: 10.1080/10408347.2023.2197062. PMID: 37029907

[52] Zhu F Zhao Q . CRISPR/Cas12a linked sandwich aptamer assay for sensitive detection of thrombin. Anal Chim Acta. (2024) 1287:342106. doi: 10.1016/j.aca.2023.342106. PMID: 38182384

[53] Hu K Yin W Bai Y Zhang J Yin J Zhu Q . CRISPR-based biosensors for medical diagnosis: Readout from detector-dependence detection toward naked eye detection. Biosensors. (2024) 14:367. doi: 10.3390/bios14080367. PMID: 39194596 PMC11353133

[54] Xu Y Ma J Dai C Mao Z Zhou Y . CRISPR/Cas12a-drived electrochemiluminescence and fluorescence dual-mode magnetic biosensor for sensitive detection of Pseudomonas aeruginosa based on iridium(III) complex as luminophore. Biosens Bioelectron. (2024) 264:116678. doi: 10.1016/j.bios.2024.116678. PMID: 39154508

[55] Vealan K Joseph N Alimat S Karumbati AS Thilakavathy K . Lateral flow assay: A promising rapid point-of-care testing tool for infections and non-communicable diseases. Asian BioMed (Res Rev News). (2023) 17:250–66. doi: 10.2478/abm-2023-0068. PMID: 38161347 PMC10754503

[56] Majdinasab M Badea M Marty JL . Aptamer-based lateral flow assays: Current trends in clinical diagnostic rapid tests. Pharm (Basel). (2022) 15. doi: 10.3390/ph15010090. PMID: 35056148 PMC8781427

[57] Kakkar S Gupta P Singh Yadav SP Raj D Singh G Chauhan S . Lateral flow assays: Progress and evolution of recent trends in point-of-care applications. Mater Today Bio. (2024) 28:101188. doi: 10.1016/j.mtbio.2024.101188. PMID: 39221210 PMC11364909

[58] Tang Y Qi L Liu Y Guo L Zhao R Yang M . CLIPON: A CRISPR-enabled strategy that turns commercial pregnancy test strips into general point-of-need test devices. Angew Chem Int Ed. (2022) 61:e202115907. doi: 10.1002/ange.202115907. PMID: 35064613

[59] Zhou J Ren X Wang X Li Z Xian CJ . Recent advances and challenges of the use of the CRISPR/Cas system as a non-nucleic acid molecular diagnostic. Heliyon. (2023) 9. doi: 10.1016/j.heliyon.2023.e22767. PMID: 38076202 PMC10703615

[60] Jiang W Zhu T Zhou S Pan L Qiao Z Wang M . Recent advances in electrochemical-based CRISPR/Cas biosensing for nucleic acid and non-nucleic acid pathogenic microorganism detection. Food Res Int. (2025) 221:117213. doi: 10.1016/j.foodres.2025.117213. PMID: 41606937

[61] Razavi Z Soltani M Pazoki-Toroudi H Chen P . CRISPR-microfluidics nexus: Advancing biomedical applications for understanding and detection. Sensors Actuators A Phys. (2024) 376:115625. doi: 10.1016/j.sna.2024.115625. PMID: 38826717

[62] Sahel DK Giriprasad G Jatyan R Guha S Korde A Mittal A . Next-generation CRISPR/Cas-based ultrasensitive diagnostic tools: Current progress and prospects. RSC Adv. (2024) 14:32411–35. doi: 10.1039/d4ra04838e. PMID: 39403159 PMC11472282

[63] Huang T Zhang R Li J . CRISPR-Cas-based techniques for pathogen detection: Retrospect, recent advances, and future perspectives. J Adv Res. (2023) 50:69–82. doi: 10.1016/j.jare.2022.10.011. PMID: 36367481 PMC10403697

[64] Hwang C Lee WJ Kim SD Park S Kim JH . Recent advances in biosensor technologies for point-of-care urinalysis. Biosens-Basel. (2022) 12. doi: 10.3390/bios12111020. PMID: 36421138 PMC9688579

[65] Lawrence SR Shah KM . Prospects and current challenges of extracellular vesicle-based biomarkers in cancer. Biol (Basel). (2024) 13. doi: 10.3390/biology13090694. PMID: 39336121 PMC11428408

[66] Masson J-F . Consideration of sample matrix effects and “biological” noise in optimizing the limit of detection of biosensors. ACS Sens. (2020) 5:3290–2. doi: 10.1021/acssensors.0c02254. PMID: 33233896

[67] Lutomia D Poria R Kala D Singh AK Gupta MK Kumar D . Unlocking the potential of 2D nanomaterial-based biosensors in biomarker-based detection of Helicobacter pylori. Mater Adv. (2024) 6:117–42. doi: 10.1039/d4ma00546e

[68] Lukman Y Shamsuddin SH . Advances in aptamer-based biosensors for cancer biomarker detection: A systematic review of emerging technologies and translational potential. Microchem J. (2026) 221:116787. doi: 10.1016/j.microc.2025.116787. PMID: 38826717

[69] Bright K Voiculescu I Penkova AN Untaroiu A . A review of biosensors and their applications. ASME Open J Eng. (2023) 2:020201. doi: 10.1115/1.4063500. PMID: 38635229

[70] Xin X Su J Cui H Wang L Song S . Recent advances in clustered regularly interspaced short palindromic repeats/CRISPR-associated proteins system-based biosensors. Biosens-Basel. (2025) 15. doi: 10.3390/bios15030155. PMID: 40136952 PMC11939850

[71] He Y Hu Q San S Kasputis T Splinter MGD Yin K . CRISPR-based biosensors for human health: A novel strategy to detect emerging infectious diseases. Trends Analyt Chem. (2023) 168. doi: 10.1016/j.trac.2023.117342. PMID: 37840598 PMC10571337

[72] Xie S Yue Y Yang F . Recent advances in CRISPR/Cas system-based biosensors for the detection of foodborne pathogenic microorganisms. Micromachines (Basel). (2024) 15. doi: 10.3390/mi15111329. PMID: 39597141 PMC11596558

[73] Sun Y Wen T Zhang P Wang M Xu Y . Recent advances in the CRISPR/Cas-based nucleic acid biosensor for food analysis: A review. Foods. (2024) 13. doi: 10.3390/foods13203222. PMID: 39456285 PMC11507162

[74] Urabe F Yamada Y Yamamoto S Tsuzuki S Kimura S Ochiya T . Extracellular vesicles and prostate cancer management: A narrative review. Transl Androl Urol. (2024) 13:442–53. doi: 10.21037/tau-23-533. PMID: 38590964 PMC10999020

[75] Safenkova IV Kamionskaya MV Sotnikov DV Biketov SF Zherdev AV Dzantiev BB . Advancing lateral flow detection in CRISPR/Cas12a systems through rational understanding and design strategies of reporter interactions. Biosens-Basel. (2025) 15. doi: 10.3390/bios15120812. PMID: 41440293 PMC12730283

[76] Guo J Wei T Huang Q Li M Yang C Mou J . Direct acupuncture of nitric oxide by an electrochemical microsensor with high time-space resolution. Biosens Bioelectron. (2022) 195:113667. doi: 10.1016/j.bios.2021.113667. PMID: 34598107

[77] Erdbrügger U Blijdorp CJ Bijnsdorp IV Borràs FE Burger D Bussolati B . Urinary extracellular vesicles: A position paper by the Urine Task Force of the International Society for Extracellular Vesicles. J Extracell Vesicles. (2021) 10:e12093. doi: 10.1002/jev2.12093, PMID: 34035881 PMC8138533

[78] Théry C Witwer KW Aikawa E Alcaraz MJ Anderson JD Andriantsitohaina R . Minimal information for studies of extracellular vesicles 2018 (MISEV2018): A position statement of the International Society for Extracellular Vesicles and update of the MISEV2014 guidelines. J Extracell Vesicles. (2018) 7:1535750. doi: 10.1080/20013078.2018.1535750, PMID: 30637094 PMC6322352

[79] Welsh JA Goberdhan DCI O'Driscoll L Buzas EI Blenkiron C Bussolati B . Minimal information for studies of extracellular vesicles (MISEV2023): From basic to advanced approaches. J Extracell Vesicles. (2024) 13:e12404. doi: 10.1002/jev2.12404. PMID: 38326288 PMC10850029

[80] Lourenço C Constâncio V Henrique R Carvalho Â Jerónimo C . Urinary extracellular vesicles as potential biomarkers for urologic cancers: An overview of current methods and advances. Cancers (Basel). (2021) 13. doi: 10.3390/cancers13071529, PMID: 33810357 PMC8036842

[81] Eskra JN Rabizadeh D Pavlovich CP Catalona WJ Luo J . Approaches to urinary detection of prostate cancer. Prostate Cancer Prostatic Dis. (2019) 22:362–81. doi: 10.1038/s41391-019-0127-4. PMID: 30655600 PMC6640078

[82] Yuan F Li YM Wang Z . Preserving extracellular vesicles for biomedical applications: Consideration of storage stability before and after isolation. Drug Delivery. (2021) 28:1501–9. doi: 10.1080/10717544.2021.1951896. PMID: 34259095 PMC8281093

[83] Barreiro K Dwivedi OP Valkonen S Groop PH Tuomi T Holthofer H . Urinary extracellular vesicles: Assessment of pre-analytical variables and development of a quality control with focus on transcriptomic biomarker research. J Extracell Vesicles. (2021) 10:e12158. doi: 10.1002/jev2.12158. PMID: 34651466 PMC8517090

[84] López-Guerrero JA Valés-Gómez M Borrás FE Falcón-Pérez JM Vicent MJ Yáñez-Mó M . Standardising the preanalytical reporting of biospecimens to improve reproducibility in extracellular vesicle research - A GEIVEX study. J Extracell Biol. (2023) 2:e76. doi: 10.1002/jex2.76. PMID: 38939690 PMC11080825

[85] Jain G Das P Ranjan P Neha Valderrama F Cieza-Borrella C . Urinary extracellular vesicles miRNA—A new era of prostate cancer biomarkers. Front Genet. (2023) 14:2023. doi: 10.1007/978-1-60761-685-6_6. PMID: 36741322 PMC9895092

[86] Haese A Trooskens G Steyaert S Hessels D Brawer M Vlaeminck-Guillem V . Multicenter optimization and validation of a 2-gene mRNA urine test for detection of clinically significant prostate cancer before initial prostate biopsy. J Urol. (2019) 202:256–63. doi: 10.1097/ju.0000000000000293. PMID: 31026217

[87] Jain G Das P Ranjan P Neha Valderrama F Cieza-Borrella C . Urinary extracellular vesicles miRNA-A new era of prostate cancer biomarkers. Front Genet. (2023) 14:1065757. doi: 10.1007/978-1-60761-685-6_6. PMID: 36741322 PMC9895092

[88] Leyten GH Hessels D Jannink SA Smit FP de Jong H Cornel EB . Prospective multicentre evaluation of PCA3 and TMPRSS2-ERG gene fusions as diagnostic and prognostic urinary biomarkers for prostate cancer. Eur Urol. (2014) 65:534–42. doi: 10.1016/j.eururo.2012.11.014. PMID: 23201468

[89] Chang EK Gadzinski AJ Nyame YA . Blood and urine biomarkers in prostate cancer: Are we ready for reflex testing in men with an elevated prostate-specific antigen? Asian J Urol. (2021) 8:343–53. doi: 10.1016/j.ajur.2021.06.003. PMID: 34765442 PMC8566358

[90] Lange S Bernstein DE Dimov N Puttaswamy S Johnston I Kraev I . Urinary extracellular vesicle signatures as biomarkers in prostate cancer patients. Int J Mol Sci. (2025) 26:6895. doi: 10.3390/ijms26146895. PMID: 40725142 PMC12295355

[91] Eklund M Jäderling F Discacciati A Bergman M Annerstedt M Aly M . MRI-targeted or standard biopsy in prostate cancer screening. N Engl J Med. (2021) 385:908–20. doi: 10.1056/nejmoa2100852. PMID: 34237810

[92] Guo S Jiang X . Personalized prostate biopsy protocols: Enhancing cancer detection through tailored approaches-a narrative review. Transl Androl Urol. (2025) 14:831–40. doi: 10.21037/tau-24-619. PMID: 40226054 PMC11986550

[93] Hessels D Schalken JA . Urinary biomarkers for prostate cancer: A review. Asian J Androl. (2013) 15:333–9. doi: 10.1038/aja.2013.6. PMID: 23524531 PMC3739649

[94] Erdmann K Distler F Gräfe S Kwe J Erb HHH Fuessel S . Transcript markers from urinary extracellular vesicles for predicting risk reclassification of prostate cancer patients on active surveillance. Cancers (Basel). (2024) 16. doi: 10.3390/cancers16132453. PMID: 39001515 PMC11240337

